# Molecular Interactions of Resistant Dextrin with Wheat Starch and Gluten: Structural Dynamics and Macromolecular Network Formation

**DOI:** 10.3390/foods15101620

**Published:** 2026-05-07

**Authors:** Yue Sun, Lu Wang, Yinta Li, Xue Bai, Rui Yang, Lili Wang, Ruge Cao

**Affiliations:** 1 College of Food Science and Engineering, Tianjin University of Science and Technology, Tianjin 300457, China; 2 Weihai Key Laboratory of Medical Functional Food Processing Technology, Weihai Ocean Vocational College, Weihai 264300, China; 3 Institute of Food Science and Technology, Chinese Academy of Agricultural Sciences, Ministry of Agriculture, Beijing 100193, China; 4 Institute of Food and Nutrition Development, Ministry of Agriculture and Rural Affairs, Beijing 100081, China

**Keywords:** resistant dextrin, starch–gluten interaction, macromolecular structure

## Abstract

Refined wheat staple foods are widely criticized for low dietary fiber and high postprandial glycemic response, making soluble dietary fiber fortification a promising strategy for cereal improvement. This study investigated how resistant dextrin (RD) modulates wheat starch, gluten, dough, and bread quality through multiscale interactions. In wheat starch, 6% RD gave the best overall balance, reducing 14-day retrogradation from 57.2% to 48.6%, delaying gelatinization, and restricting amylose diffusion, with hydrogen bonding identified as a major contributing interaction. In gluten, RD increased water-holding capacity but weakened network integrity, as evidenced by reduced moduli, a shift in thiol–disulfide balance, secondary-structure redistribution (increased β-sheet, decreased α-helix/β-turn), and suppressed glutenin polymerization, yielding a looser microstructure. In dough, SEM and rheological results suggested that moderate RD (4–6%) may form a hydrated, polysaccharide-rich phase that fills structural voids and improves matrix continuity, partially offsetting gluten weakening and enhancing viscoelasticity. Overall, this study establishes a quantitative relationship between RD addition level, multiscale macromolecular interactions in wheat matrices, and the processing performance and quality of bakery products.

## 1. Introduction

Wheat flour-based foods remain central to human diets, yet their nutritional and technological performance is largely governed by starch, which typically accounts for nearly 60–75% of flour dry mass [1]. In refined wheat products, this starch-rich composition promotes rapid enzymatic hydrolysis and glucose release, contributing to elevated postprandial glycemic responses [2]. Bread is the most widely consumed wheat-based staple worldwide, accounting for more than 60% of wheat flour product consumption in most regions [3]. It is a major source of dietary carbohydrates and an important carrier for fiber fortification. However, refined commercial bread is typically low in dietary fiber and has a high postprandial glycemic response, and it also stales rapidly after baking, which limits both its nutritional value and market performance [4]. These concerns have intensified interest in reformulating wheat products toward higher fiber content and reduced glycemic impact [5]. However, fiber enrichment frequently impairs dough handling, gas retention and final product quality because added polysaccharides compete for free water and engage in hydrogen bonding with starch and gluten, thereby perturbing hydration equilibria and destabilizing the starch–gluten network that underlies dough viscoelasticity [6].

Resistant dextrin (RD) is a low-digestible, dextrinized starch-derived polysaccharide that has gained attention as a soluble dietary fiber for bakery applications. Its high solubility, water binding capacity, minimal flavor impact, and partial fermentability to short chain fatty acids (SCFAs) support its appeal for improving nutritional profiles without introducing strong sensory penalties [7]. Notably, these functional properties are closely related to the unique molecular architecture of RD. Unlike the linear structure of inulin, the rigid high-molecular-weight chains of β-glucan, or the randomly polymerized structure of polydextrose, RD is a highly branched dextrin with a low degree of polymerization, high hydroxyl density, and flexible chain conformation. These features determine its specific interaction behavior with wheat components, indicating that the mechanisms reported for other soluble fibers cannot be directly applied to RD [8,9].

The processing performance of wheat dough emerges from multiscale interactions among starch granules, gluten proteins (gliadin and glutenin), and water [10]. Soluble polysaccharides can modify this system through two coupled routes. First, by competing for water and altering its mobility, they shift hydration of starch and proteins, thereby influencing starch gelatinization/pasting, protein plasticization, and the kinetics of starch retrogradation [11,12]. Second, they may associate with starch chains and gluten polypeptides through non-covalent interactions, predominantly hydrogen bonding, with possible contributions from hydrophobic contacts, thereby altering chain packing, protein conformation, and network connectivity [13]. Prior work has documented changes in pasting profiles, viscoelastic moduli, and baking outcomes after soluble fiber addition. However, many studies emphasize macroscopic endpoints and do not resolve how RD-driven molecular interactions propagate to mesoscale structure. They also do not fully explain how these changes ultimately affect dough rheology and product quality.

Nevertheless, several important questions regarding RD remain unresolved. First, most previous studies have focused on starch or gluten alone, and systematic evidence linking RD regulation across starch, gluten, and the integrated dough–bread system from molecular to macroscopic levels is still limited [14]. Second, the main intermolecular forces involved in RD–wheat interactions remain largely inferred, with little direct experimental verification from chemical perturbation rheology [15]. Third, the trade-off effect of RD, namely its ability to inhibit starch retrogradation while simultaneously weakening the gluten network, has not been fully clarified. In particular, the mechanism by which moderate RD can offset gluten weakening and still preserve product quality remains unclear [16].

In this study, pasting and thermal analyses are therefore integrated with targeted spectroscopic measurements, microstructural imaging, and dynamic rheology to examine how graded RD incorporation reshapes wheat starch (WS) gelatinization/retrogradation, network development of gluten protein (GP), and the integrated viscoelastic response of wheat dough, alongside baking performance. By linking molecular- and mesoscale-level changes to rheological signatures and quality attributes, these criteria support nutritionally improved wheat products without sacrificing processing robustness or consumer relevant texture.

## 2. Materials and Methods

### 2.1. Reagents and Chemicals

Citric acid, transglucosidase and urea were purchased from Shanghai Aladdin Biochemical Technology Co., Ltd. (Shanghai, China). The α-amylase (from *Bacillus licheniformis*) was obtained from Tokyo Chemical Industry Co., Ltd. (Tokyo, Japan). Potato amylose was supplied by Tianjin Bohua Chemical Reagent Co., Ltd. (Tianjin, China). Absolute ethanol, sodium hydroxide (NaOH), hydrochloric acid (HCl) and Tris-HCl were sourced from Tianjin Jiangtian Chemical Technology Co., Ltd. (Tianjin, China).

### 2.2. Preparation of RD and Related Experimental Samples

RD was prepared through a modified thermal acid treatment method following enzymatic hydrolysis of corn starch [17,18]. Briefly, 100 g of corn starch was mixed with citric acid at 1.0% (*w*/*w*, based on the dry mass of starch). The mixture was stirred at room temperature until a viscous paste formed, then dried, milled, and passed through a 100-mesh sieve. The resulting powder was subsequently subjected to thermal treatment at 160–190 °C [19]. The treated powder was dispersed in distilled water (1:5 *w*/*w*), pH-adjusted to 5.5, and hydrolyzed with thermostable α-amylase at 95 °C (with subsequent boiling to inactivate the enzyme). Next, the sample was reacted with transglucosidase at 60 °C followed by a second heat inactivation. After cooling, three volumes of absolute ethanol were added to precipitate the product, which was collected by centrifugation (4000× *g*, 10 min), dried, milled and reconstituted as a 40% (*w*/*v*) solution. This solution was decolorized with activated carbon, dialyzed, concentrated and lyophilized to yield the final RD (water solubility 93.8%, resistant content 77.3%).

To evaluate the effect of different addition levels of RD on WS, wheat starch and resistant dextrin mixtures (WS-RD) were prepared by homogenizing WS with RD at addition levels of 0%, 2%, 4%, 6%, 8%, and 10% in 20 mL distilled water with continuous stirring for 10 min. The suspensions were gelatinized in a 95 °C water bath for 20 min and then allowed to equilibrate to 25 °C before further measurements.

To investigate the effect of different addition levels of RD on the GP of wheat, gluten protein and resistant dextrin mixtures (GP-RD) were prepared. The same dry-basis addition strategy was used as in the WS-RD system. Briefly, the dry mass of vital wheat gluten was kept constant across all groups, and RD was added at 0%, 2%, 4%, 6%, 8%, and 10% (*w*/*w*, based on the dry mass of gluten). Vital GP was uniformly blended with RD at 0–10% (*w*/*w*) addition levels. The composite powders (200 g) were hydrated with distilled water (60% flour basis) and kneaded to optimal dough development (farinograph consistency index > 1.1 N·m) using a spiral mixer (DL-6000, Diosna, Osnabrück, Germany). The resultant dough was lyophilized, pulverized, and sieved (100 mesh) to obtain homogeneous RD–gluten complexes.

GP extraction was performed according to the protocol of Bao et al. [20] with optimization. Briefly, 1 g of composite powder was suspended in 5 mL of protein extraction buffer containing 7 mol/L urea, 50 mmol/L dithiothreitol (DTT), and 2 mol/L thiourea (pH 8.8) with ultrasonication (40 kHz, 300 W) at 60 °C for 30 min. After centrifugation (12,000× *g*, 15 min), the resulting supernatant was collected as the total protein extract and subsequently freeze-dried to obtain the GP-RD powder for further analysis.

### 2.3. Characterization of WS-RD Mixtures

#### 2.3.1. Rapid Viscosity Analysis (RVA)

The pasting characteristics were evaluated using a Rapid Viscosity Analyzer (RVA, Rapid-20, Bosin Tech, Shanghai, China) following the method of Cheng et al. [21] with modifications. Briefly, 2.5 g WS-RD mixtures (dry basis, with RD addition levels of 0–10% *w*/*w*) were hydrated with distilled water to achieve a total mass of 28 g in RVA aluminum canisters. The pasting procedure involved maintaining the temperature at 50 °C for 1 min, heating to 95 °C at a rate of 12 °C/min, holding at 95 °C for 3.5 min, cooling back to 50 °C at the same rate, and then holding at 50 °C for another 2 min. All measurements were performed in triplicate.

#### 2.3.2. Leached Amylose Content

The leached amylose content was quantified according to the method of Li et al. [22] with adaptations. Gelatinized WS-RD samples were centrifuged at 20,000× *g* for 30 min; then, 1 mL supernatant was alkalinized with 6 mL 0.33 M NaOH solution and incubated at 95 °C for 30 min. Subsequently, 0.1 mL hydrolysate was mixed with 5 mL 0.5% chloroacetic acid solution (pH adjusted to 5.5) and stained with I_2_-KI solution (0.01 mol/L) for 20 min. Absorbance at 620 nm was measured against distilled water blank, with reagent-grade potato amylose serving as the calibration standard. A standard curve was established for the quantitation of leached amylose content. All measurements were performed in triplicate.

#### 2.3.3. Differential Scanning Calorimetry (DSC)

The thermal properties of WS-RD mixtures were investigated using DSC (Norwalk, CT, USA) following the methodology of Zhang et al. [23] with slight modifications. Precisely 20 mg of each WS-RD formulation containing 20% (*w*/*w*) starch was hermetically sealed in aluminum pans and equilibrated at ambient temperature for 12 h. The thermal program involved heating from 20 °C to 90 °C at 10 °C/min under nitrogen atmosphere (50 mL/min). Characteristic parameters including onset (*T_o_*), peak (*T_p_*), and conclusion (*T_c_*) temperatures, along with enthalpy, were determined. All enthalpy values were normalized to the actual mass of WS in the system. To assess retrogradation, the gelatinized samples were stored at 4 °C for 3, 7, and 14 days. Before rescanning, all samples were equilibrated at room temperature for 1 h. Thermal rescans were then carried out from 4 °C to 140 °C at 10 °C/min. All measurements were performed in triplicate. The relevant thermal characteristic parameters are summarized in Table 1.

#### 2.3.4. Particle Size Distribution and Confocal Laser Scanning Microscopy (CLSM)

The particle size distribution of starch granules in WS-RD mixtures was determined using a Mastersizer 2000 (Malvern Instruments, Malvern, UK) equipped with a Hydro 2000MU dispersion unit; distilled water was used as the dispersant [24].

Microstructural evolution during gelatinization was visualized using high-resolution confocal microscopy (N-SIM, Nikon, Tokyo, Japan) [25]. Samples were prepared by incubating 100 μL WS-RD suspensions with 20 μL fluorescein isothiocyanate solution (2 mg/mL in PBS, pH 7.4) for 24 h at 20 °C. Image acquisition was performed using 488 nm excitation/515 nm emission parameters with a 60× oil-immersion objective (NA 1.4). Z-stack reconstructions were processed using NIS-Elements AR software (v4.3). The observations focused on the overall swelling, integrity, and disintegration behavior of the granules rather than the precise localization of starch molecules. All measurements were performed in triplicate.

#### 2.3.5. FT-IR

Spectra were recorded on a Nicolet iS10 FT-IR spectrometer (Thermo Fisher Scientific, Madison, WI, USA) at a resolution of 4 cm^−1^ over the range of 4000–500 cm^−1^, using 64 scans. The 1200–900 cm^−1^ region associated with starch short-range ordered structure was analyzed by second-derivative transformation and Gaussian deconvolution. The intensity ratios of 1047/1022 cm^−1^ and 995/1022 cm^−1^ were calculated to evaluate short-range order in starch.

#### 2.3.6. Molecular Interaction Assessment

Interfacial interactions between WS and RD were evaluated as described previously [26]. The experiment was performed only on the WS-RD dispersion containing 6% RD. Briefly, the dispersion was prepared in 50 mL distilled water and divided into equal aliquots, which were supplemented with NaCl and urea to final concentrations of 0, 0.1, 0.2, and 0.3 M. After thermal treatment (95 °C, 20 min) and cooling, dynamic oscillatory rheometry was performed using a DHR-2 rheometer (TA Instruments, New Castle, DE, USA) at 1% strain (within the linear viscoelastic region) with frequency sweeps from 0.1 to 10 Hz to monitor the evolution of storage modulus (*G*′) and loss modulus (*G*″). All measurements were performed in triplicate.

### 2.4. Characterization of GP-RD Mixtures

#### 2.4.1. Microstructural Characterization

RD-GP samples were frozen at −80 °C, lyophilized for 24 h, and examined by scanning electron microscopy (Hitachi S-3400, Ibaraki, Japan) at an accelerating voltage of 15 kV to observe cross-sectional morphology [27]. All measurements were performed in triplicate.

#### 2.4.2. Free Sulfhydryl (SH_free_) and Disulfide Bond (S-S) Quantification

The contents of SH_free_ groups and S-S in the RD-GP samples were determined [28]. All measurements were performed in triplicate. Briefly, 100 mg samples were reacted with Ellman’s reagent for 10 min before centrifugation (16,000× *g*, 6 min). Absorbance of the supernatant was measured at 412 nm, and free SH content (μmol/g protein) was calculated using the molar absorption coefficient ε = 13,600 M^−1^·cm^−1^.

Total sulfhydryls (SH_total_) were measured after disulfide reduction with 40 mM dithioerythritol (DTE) in 80 mM Tris/HCl (pH 8.5, 60 °C, 2 h), followed by acetone precipitation (−18 °C) and triple washing. Reduced samples were reacted with 5.5 mL Ellman’s reagent for SH_total_ determination. S-S content was calculated as

(1) SS = (SH_total_ − SH_free_)/2

#### 2.4.3. Secondary Structure and Components Analysis

The secondary structural composition of RD-GP was characterized using FTIR spectroscopy. Spectral acquisition was performed in the 4000–400 cm^−1^ wavenumber range with 4 cm^−1^ resolution and 64 cumulative scans to ensure an optimal signal-to-noise ratio. The amide I region (1600–1700 cm^−1^), which is particularly sensitive to protein conformational changes, was subjected to Fourier self-deconvolution using a Lorentzian line shape with a bandwidth of 25 cm^−1^ and an enhancement factor of 2.0. This approach is widely used for quantitative analysis of protein secondary structure. The amide I region is sensitive to conformational changes, and its band components can be resolved by spectral deconvolution. Quantitative secondary-structure composition was obtained by Gaussian curve fitting of the deconvoluted spectra using Omnic 8.0 software (Thermo Scientific) [29], with band assignments listed in Table 2.

GP-RD solutions (3 mg/mL) were mixed with an equal volume of loading buffer containing 0.4% SDS, 12% glycerol, 50 mM Tris-HCl (pH 6.8), 2% β-mercaptoethanol, and 0.01% bromophenol blue. The mixture was heated in a boiling water bath for 1 min, cooled to room temperature, and centrifuged at 4000× *g* for 10 min. The supernatant was collected for electrophoresis. SDS-PAGE was performed using a 12% resolving gel [30] on a Mini-PROTEAN Tetra system (Bio-Rad, Hercules, CA, USA). Samples were run at a constant voltage of 75 V for approximately 2.5 h, until the bromophenol blue front migrated to within 1 cm of the bottom of the gel. Upon completion, the gel was immersed in staining solution and agitated for 30 min, followed by repeated destaining until a clear protein band pattern was achieved. All measurements were performed in triplicate.

#### 2.4.4. Intrinsic Fluorescence Spectroscopy

The intrinsic fluorescence characteristics of RD-GP were evaluated based on previous work [31]. Protein solutions were prepared by dissolving 50 mg of lyophilized gluten samples in 5 mL phosphate buffer (10 mM, pH 7.0), followed by vortex mixing (30 s) and centrifugation (4000× *g*, 15 min) to obtain clarified supernatants. Fluorescence emission spectra (300–500 nm) were recorded on a spectrofluorometer (F-7000, Hitachi, Tokyo, Japan) using 280 nm excitation with both excitation and emission slit widths set at 5 nm. The intrinsic fluorescence intensity and spectral characteristics of the protein samples were recorded and analyzed to assess conformational changes and microenvironmental variations in aromatic residues. All measurements were performed in triplicate.

#### 2.4.5. Water- and Oil-Holding Capacity

The water-holding capacity (WHC) and oil-holding capacity (OHC) of RD-GP were determined based on the method reported by Zhao et al. [32]. All measurements were performed in triplicate. A 1.0 g sample (m_0_) was weighed into pre-tared centrifuge tubes (m_1_), hydrated with 20 mL distilled water, and vortex-mixed thoroughly. After 30 min equilibration, samples were centrifuged (3000× *g*, 15 min), supernatants decanted, and pellets carefully blotted with filter paper before reweighing (m_2_). WHC was calculated as

(2) WHC (g/g) = (m_2_ − m_1_)/m_0_

where m_0_ refers to the mass of the dried sample; m_1_ refers to the mass of the centrifuge tube; m_2_ refers to the mass of the centrifuge tube and precipitate.

OHC measurement employed identical procedures substituting distilled water with 10 mL rapeseed oil, incorporating 1 min vortex mixing followed by 30 min static incubation prior to centrifugation.

(3) OHC (g/g) = (m_2_ − m_1_)/m_0_

### 2.5. Preparation and Evaluation of RD Enriched Products

#### 2.5.1. Textural Properties

Wheat flour was thoroughly blended with RD at addition levels of 0%, 2%, 4%, 6%, 8%, and 10% (*w*/*w*). Precisely 200 g of each premix was mixed with distilled water. The mixture was kneaded in a dough mixer until a smooth and homogeneous RD-fortified dough was obtained. The dough was then lyophilized, milled, and passed through a sieve to yield uniform dextrin–dough powders [33].

Dough extensibility was measured following Song et al. [34]. The proofed dough is kneaded into strips and fixed on a Kieffer stretching fixture. The middle hook then pulls the dough upward at a constant speed until it breaks. Then, the software automatically records and outputs the distance when it breaks, which is the ductility. All measurements were performed in triplicate.

Bread was prepared on the basis of the dough with different RD addition levels. The dough rested for 20 min at 30 °C and fermented for 60 min, then degassed and molded. Proofing and baking were done under set conditions, and the bread was cooled before analysis [35]. Bread were sectioned into 2 cm^3^ cubes. Texture analysis was performed using a texture analyzer (TA-XT Plus, Stable Micro Systems, Godalming, UK) equipped with a P/36R probe in TPA mode. The test parameters were as follows: trigger force, 5 g; pre-test, test, and post-test speeds, 1.0 mm/s; and interval between the two compressions, 5 s [36]. All measurements were performed in triplicate.

#### 2.5.2. Specific Volume, and Staling Characteristics

The specific volume of bread was measured according to Shimada et al. [37]. The weight (g) was obtained with a balance, and volume (mL) was determined via rapeseed displacement. Diameter (cm) and height (cm) were measured with vernier calipers. Specific volume was calculated using following equation.

(4) Specific volume (mL/g) = Volume/Weight

The hardness and elasticity of bread crumbs were monitored at storage intervals of 0, 1, 3, 7, and 14 days. Crumb samples (2 cm^3^ cubes) were subjected to TPA analysis under identical instrumental parameters as described in Section 2.5.1. For each storage time point, six replicates were measured, with aberrant values discarded before statistical computation.

### 2.6. Statistical Analysis

All results were expressed as the mean ± standard deviation (SD) of at least three replicates. Statistical analysis was conducted using SPSS software (version 26.0, IBM, Armonk, NY, USA). Significant differences between means were determined by Duncan’s multiple range test at *p* < 0.05.

## 3. Results and Discussion

RD-enabled reformulation of wheat products remains largely empirical because functionality emerges from multiscale interactions among starch, gluten, and water, with outcomes strongly dependent on RD type and dosage. Here, graded RD incorporation was systematically examined in WS, gluten, and the integrated dough/bread system. Across measurements, RD exhibited a clear dosage window, with intermediate addition levels providing the most favorable balance between structural stabilization and network continuity.

### 3.1. Effect of RD on WS Properties

The RD used in this study was obtained from corn starch through thermal acid degradation and subsequent repolymerization, resulting in the loss of native starch granule structure. Therefore, its interaction with WS is governed primarily by its intrinsic molecular features (e.g., high hydroxyl density, flexible chain conformation, and strong hydration capacity), rather than by the botanical origin of the precursor starch.

#### 3.1.1. Pasting Behavior and Amylose Leaching

**Figure 1:**
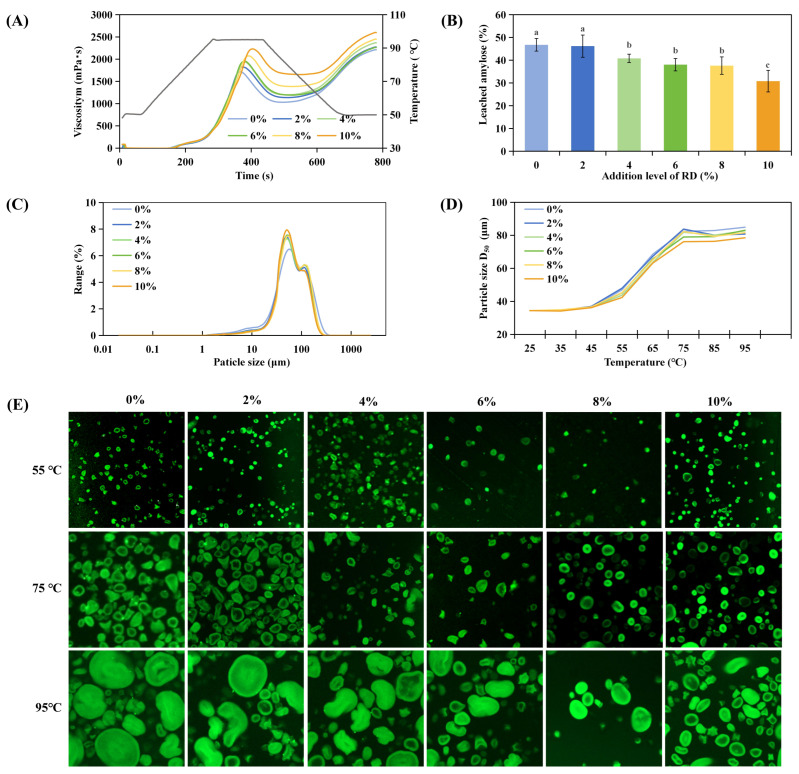
Effects of resistant dextrin (RD) on the pasting behavior and granular structure of wheat starch (WS). (**A**) Pasting viscosity curves, (**B**) leached amylose content, (**C**) particle size distribution, (**D**) median particle size D_50_, (**E**) CLSM images of starch granules at 55, 75 and 95 °C in WS-RD system. Different superscript letters in the same column represents significant difference (*p* < 0.05).

RD increased the thermal requirement for WS gelatinization and reshaped viscosity evolution during heating (Figure 1A; Table 1). The grey line presents the temperature program of the test. It started from an initial temperature of 50 °C, rose to 95 °C at a heating rate of 10 °C/min, held at 95 °C, and then cooled down to 50 °C over the total 800 s test period. As RD increased from 0% to 10%, the pasting temperature (PT) rose from 88.1 °C to 89.7 °C, accompanied by ~3 °C increases in DSC peak and terminal temperatures, together with an increase in gelatinization enthalpy (Δ*H*_1_).

These coordinated shifts indicate delayed swelling and gelatinization in the presence of RD, which is likely attributable to high solubility and strong water-binding capacity that reduce free-water availability and mobility for granule hydration [38]. Because Δ*H*_1_ is sensitive to hydration state and bound-water distribution, the combined temperature and enthalpy changes support a hydration-mediated constraint on granule disordering rather than a single-parameter effect.

RVA parameters (Table 1) further reflected RD-induced constraints on swelling and shear response. Peak (PV) and trough viscosities (TVs) increased with RD addition, while breakdown viscosity increased up to 6% RD and then decreased, indicating a non-linear response. Moderate RD enhanced apparent paste viscosity and stabilized swollen structures under shear, whereas higher RD increasingly restricted granule swelling and chain mobility. These effects arise from intensified water competition and medium thickening. Setback viscosity decreased by nearly 14% at 10% RD, indicating suppressed short-term reassociation during cooling. The observed reduction in retrogradation is consistent with restricted molecular mobility and steric hindrance, which limit amylose alignment and recrystallization [39,40].

Amylose leaching directly confirmed RD-induced restriction of starch chain diffusion during gelatinization (Figure 1B). Leached amylose decreased from 46.78% (control) to 30.82% at 10% RD. This reduction reflects limited amylose release and diffusion, plausibly due to increased continuous-phase viscosity and a denser hydrogen-bonded polysaccharide environment.

During storage, RD inhibited retrogradation in a dosage-dependent manner; retrogradation enthalpy (Δ*H*_2_) and retrogradation degree (R) at 3, 7, and 14 days decreased with increasing RD, with maximal inhibition at 6% RD (14-day Δ*H*_2_: from 79.0 to 71.5 J/g; R: from 57.2% to 48.6%). Beyond 6%, the effect plateaued, with no significant differences between 8% and 10% treatments (*p* > 0.05). This indicates an effective dosage range beyond which further constraint yields diminishing returns. It should be noted that the proposed hydration-related mechanism is inferred mainly from pasting and thermal parameters, as water distribution and mobility in the WS–RD system were not directly measured in this study.

#### 3.1.2. Particle Size Distribution and CLSM

Particle size distribution and CLSM observations corroborated the pasting and thermal results (Figure 1C–E). All samples exhibited bimodal distributions (1–500 μm), dominated by an intermediate fraction (30–100 μm). Increasing RD reduced signals at both small (<10 μm) and large (>100 μm) sizes while enhancing the intermediate peak (Figure 1C), with median particle size decreasing at 6–10% RD (Figure 1D). These trends indicate suppression of excessive swelling and aggregation during heating, consistent with increased continuous-phase viscosity and reduced effective water availability limiting granule expansion [41].

CLSM images visualized these effects during heating (Figure 1E). At 55 °C, granules retained clear boundaries across all treatments. At 75 °C, the control showed rapid swelling and blurred contours, whereas RD-containing samples displayed constrained expansion and more regular morphologies. At 95 °C, the control showed extensive disintegration, whereas high-RD systems retained partially intact, spherical structures. This indicates that RD modulates gelatinization primarily by altering hydration and medium viscosity. Consequently, swelling and disintegration dynamics are constrained [42]. For CLSM observations, the analysis focused on overall granule swelling, integrity, and disintegration behavior. Although FITC may non-specifically bind trace residual proteins, this does not materially affect the qualitative assessment of granule morphology.

#### 3.1.3. FT-IR Spectroscopy and Interaction Mechanism

**Figure 2:**
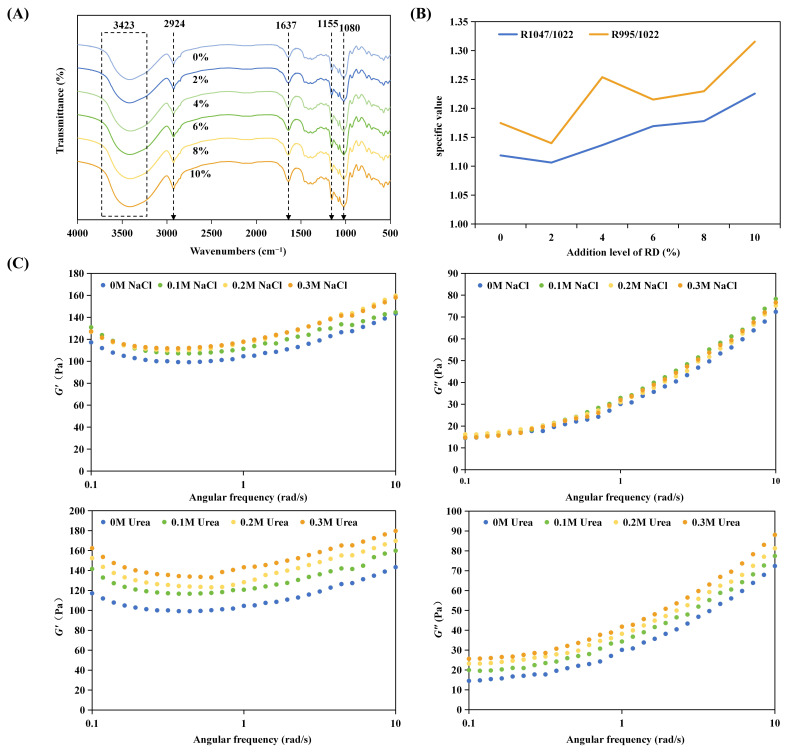
Molecular interaction analysis between wheat starch (WS) and resistant dextrin (RD). (**A**) FTIR spectra, (**B**) intensity ratios of characteristic FTIR bands (1047/1022 cm^−1^ and 995/1022 cm^−1^), and (**C**) effects of chemical perturbants (NaCl and urea) on the rheological properties of the WS-RD system.

FT-IR spectra (Figure 2A) showed systematic intensity changes at 3424 cm^−1^ (O-H stretching) and 1637 cm^−1^ (bound water) with increasing RD content. As RD itself is a hydroxyl-rich polysaccharide, its spectral features overlap with those of WS. Therefore, these changes may partly reflect additive spectral contributions from RD rather than solely interaction-induced shifts. Overall, the results indicate modifications in the hydrogen-bonding environment and water-binding state within the composite system.

Short-range order indicators supported this interpretation (Figure 2B). Increases in the 1047/1022 cm^−1^ ratio indicate enhanced local chain packing, while the rise in the 995/1022 cm^−1^ ratio (from 1.14 to 1.32) reflects strengthened association and modified local hydrogen bonding environments [43]. These indices reflect the overall short-range ordering of the WS-RD system; as long-range crystallinity was not directly assessed, the interpretation is limited to local structural organization.

To probe intermolecular interactions, chemical perturbation rheology was conducted on the 6% RD system (Figure 2C). NaCl produced negligible changes in *G*′ and *G*″, indicating minimal electrostatic contribution [44]. In contrast, urea led to a marked, concentration-dependent decrease in both moduli, with a reduction of over 75% in G′ at 0.3 M. This suggests that hydrogen bonding is a major contributing interaction in maintaining the structure of the gelatinized WS-RD system at this concentration. Taken together with thermal and pasting results, RD appears to modulate starch behavior through combined effects of hydration changes and hydrogen-bond-mediated restriction of chain mobility.

### 3.2. Effects of RD on Gluten Structure and Functionality

#### 3.2.1. Hydration Properties and Viscoelastic Behavior

**Figure 3:**
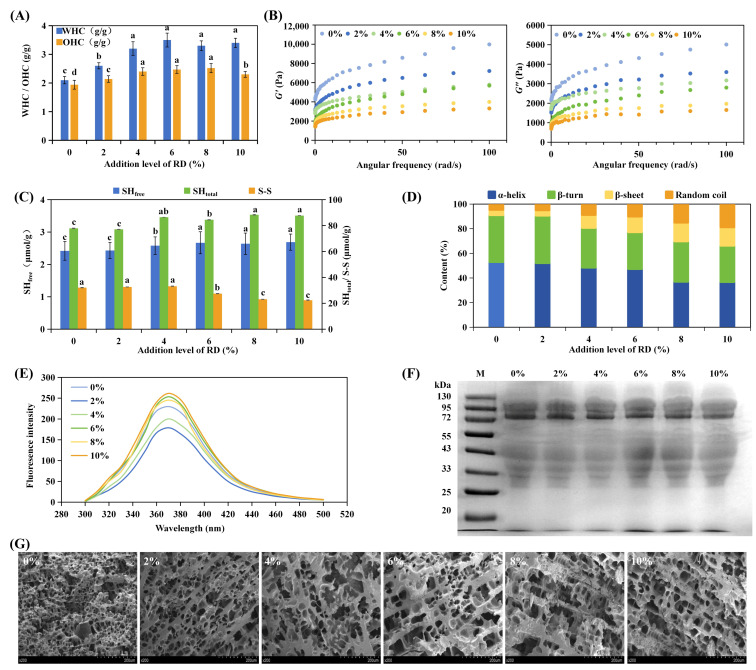
Impact of resistant dextrin (RD) on the structure and functionality of gluten. (**A**) Water- and oil-holding capacity, (**B**) dynamic rheological properties, (**C**) free sulfhydryl and disulfide bond content, (**D**) secondary structure composition, (**E**) intrinsic fluorescence spectra, (**F**) SDS-PAGE protein profile, and (**G**) scanning electron micrographs of the gluten–RD system. Different superscript letters indicate statistically significant differences among samples (*p* < 0.05).

WHC increased from low to moderate RD addition levels and then reached a plateau: samples containing 4–10% RD exhibited similarly higher WHC values than those at 0–2% (Figure 3A). This indicates that RD-enhanced water binding becomes saturated once sufficient hydroxyl-rich polysaccharide is present, and further RD addition does not yield additional water retention. OHC showed a different trend, increasing at 4–8% RD compared with 0–2%, but decreasing at 10% RD. Moderate RD levels likely loosen protein packing and expose hydrophobic regions, enhancing oil binding. However, excessive RD addition, strong hydration and polysaccharide crowding may mask hydrophobic sites or restrict oil penetration, leading to reduced OHC [45]. These results indicate that RD modulates gluten hydration and surface accessibility in a concentration-dependent manner. Moderate RD levels enhance both water and oil interactions, whereas at higher levels, excessive hydration becomes dominant.

Dynamic rheology (Figure 3B) shows that all samples remained predominantly elastic (*G*′ > *G*″), but both *G*′ and *G*″ fell progressively with increasing RD. This leads to an apparent discrepancy: RD increases the bulk water-holding capacity while simultaneously reducing the elastic modulus of gluten. This behavior suggests that increased hydration does not necessarily translate into stronger network formation. Specifically, RD competes for free water and interposes between gluten chains, increasing inter-chain spacing and reducing the density of contacts required for an elastic network [46]. Thus, RD shifts the system from a tightly cross-linked protein gel toward a more hydrated, polysaccharide-plasticized matrix with lower elastic modulus.

#### 3.2.2. S-S Bond Equilibrium and Secondary Structure

RD affected both covalent and conformational characteristics of gluten proteins. Under identical denaturing extraction conditions, S-S content decreased while free -SH groups increased with increasing RD (Figure 3C), indicating reduced disulfide re-formation during extraction. This may be associated with increased molecular separation and altered solvent environment [47]. These results should be interpreted as comparative changes in the extracted protein fraction rather than the in situ gluten network.

FT-IR analysis (Figure 3D) showed an increase in β-sheet content and a decrease in α-helix and β-turn structures with increasing RD. These changes likely reflect RD-induced alterations in hydration and intermolecular environment during extraction, promoting partial unfolding and reorganization into more extended structures. However, given the denaturing conditions, these results should be interpreted cautiously when relating to native dough structure.

#### 3.2.3. Tertiary Structure and Subunit Distribution

Intrinsic fluorescence showed a slight red shift in λ_max_ and a biphasic change in fluorescence intensity (Figure 3E): an initial quenching at low RD (2%), followed by partial recovery or increased intensity at ≥6%. The early quench is consistent with exposure of tryptophan residues to a more polar environment upon partial unfolding. The later increase plausibly reflects formation of stabilized aggregates or refolded conformers that re-shield aromatic residues or create microenvironments of reduced polarity [48]. These fluorescence trends are congruent with the observed thiol–disulfide perturbation and secondary-structure reorganization.

SDS-PAGE profiles (Figure 3F) corroborate subunit redistribution: high-molecular-weight glutenin subunits (>100 kDa) progressively weaken with increasing RD, while medium- to low-molecular-weight bands (≈33–43 kDa) become relatively more prominent. This pattern indicates suppression of glutenin polymerization and partial depolymerization or dissociation of aggregates, consistent with reduced S-S cross-linking and steric/solvent effects that limit subunit association [11].

#### 3.2.4. Microstructure and Functional Implications

SEM images (Figure 3G) show a compact, continuous network in the control and progressively looser, more porous protein matrices with RD addition. Strands become thicker and inter-strand connectivity decreases while pore size and heterogeneity increase. These morphological changes are the structural manifestation of the combined effects documented above—increased continuous-phase hydration, reduced disulfide cross-link density, and secondary-structure shifts. Together, these factors explain the observed reductions in elastic moduli.

RD redistributes water through extensive hydrogen bonding, enhancing bulk hydration while perturbing local protein hydration and sterically hindering protein–protein interactions. Consequently, gluten exhibited reduced cross-linking, a shift toward β-sheet-rich conformations, and a looser microstructure. In dough systems, these effects may be partially compensated by the presence of a hydrated polysaccharide phase.

### 3.3. Effects of RD on Wheat Dough and Bread Quality

#### 3.3.1. Microstructure and Gluten Network

**Figure 4:**
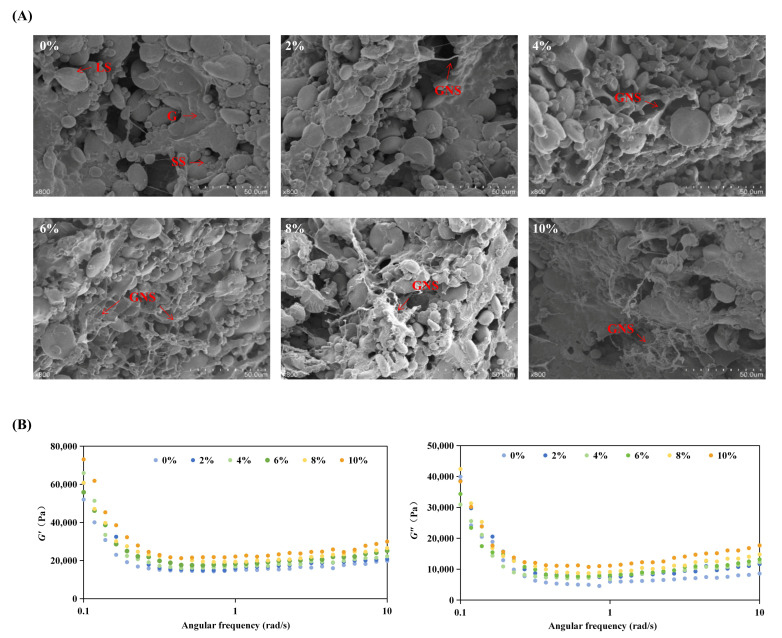
Microstructural and rheological evolution of dough with resistant dextrin (RD). (**A**) SEM images of dough microstructure: G (gluten), LS (large starch granules), SS (small starch granules), GNSs (gluten network skeletons). (**B**) Dynamic rheological properties (*G*′ and *G*″) of dough with varying RD levels. Different superscript letters indicate statistically significant differences among samples (*p* < 0.05).

SEM images of mixed dough (Figure 4A) show that the control dough presented a relatively continuous gluten matrix in which starch granules were embedded and partially swollen. After RD incorporation, the microstructure changed in an addition-dependent manner. At 2–6% RD, the dough exhibited a more compact morphology, characterized by reduced voids and a more integrated continuous phase. At RD levels ≥8%, the dough matrix became increasingly discontinuous, with more exposed starch granules and clearer phase separation, indicating weakened interfacial connectivity.

Quantitative gluten measurements (Table 3) were consistent with these observations. Wet gluten increased at 4–6% RD (*p* < 0.05 vs. 0–2%). In contrast, dry gluten decreased progressively as RD increased, indicating higher water retention within the gluten-rich fraction rather than greater protein mass. RD weakened the self-supporting gluten network when gluten was isolated, yet in real dough moderate RD improved the continuity of the multiphase system. This provides evidence for a mechanism through which RD primarily modifies the dough through hydration regulation and continuous-phase thickening: moderate RD forms a hydrated polysaccharide-rich phase that fills interstitial spaces and bridges protein–starch domains, while excessive RD competes strongly for water and increases phase heterogeneity, leading to local discontinuities and poorer starch encapsulation [49]. The proposed mechanism, that moderate RD forms a hydrated, polysaccharide-rich phase that fills structural voids, is inferred from SEM and rheological evidence. Direct visualization of such domains would require targeted imaging techniques.

#### 3.3.2. Dynamic Rheological Behavior of Dough with RD

The frequency sweep results (Figure 4B) showed that both *G*′ and *G*″ displayed stronger frequency dependence at low frequencies (<1 Hz) and a tendency toward a slower increase at higher frequencies, indicative of a structured viscoelastic system with increasing resistance to deformation under faster oscillation. In all samples, *G*′ remained higher than *G*″, confirming that elastic behavior dominated within the tested range.

**Figure 5:**
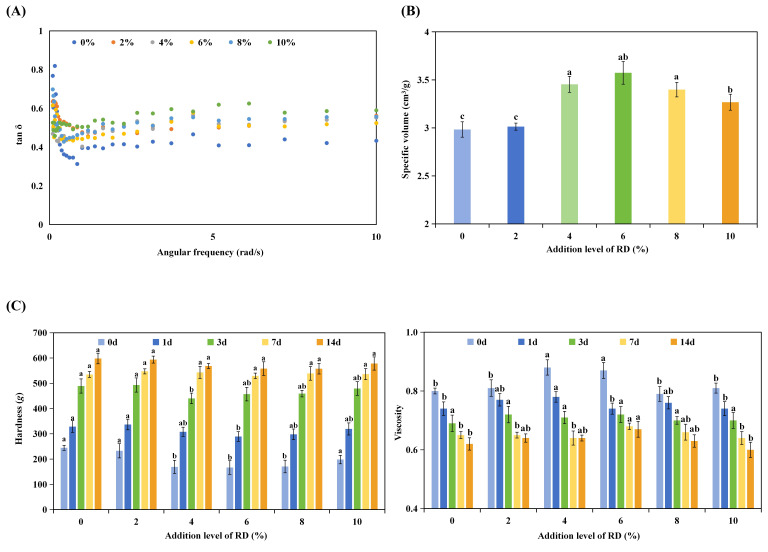
Baking performance and storage stability of bread containing resistant dextrin (RD). (**A**) Dynamic rheological properties (tan δ) of dough, (**B**) specific volume, and (**C**) changes in hardness and elasticity during storage for breads with different RD addition levels. Different superscript letters indicate statistically significant differences among samples (*p* < 0.05).

As shown in Figure 5A, tan*δ* values remained below 1 across the tested frequency range, indicating predominantly elastic behavior. A frequency-dependent trend was observed, with tan*δ* decreasing at low frequencies and increasing at higher frequencies, consistent with weak gel behavior. RD addition produced a clear dose-dependent effect. At low frequencies (<0.2 rad/s), 2–6% RD reduced tan*δ*, indicating enhanced elastic dominance under near-static conditions. At higher frequencies (0.2–10 rad/s), tan*δ* increased with RD content, suggesting a greater viscous contribution under processing-relevant shear. At ≥8% RD, this shift became more pronounced, consistent with weakened gluten structure and reduced network integrity. These results indicate that RD primarily increases viscous contributions rather than strengthening elasticity, in agreement with observations in the gluten system.

This strengthening effect is notable because isolated gluten systems in Section 3.2 showed reduced moduli with RD addition, highlighting that dough performance cannot be inferred from gluten-only behavior. In composite dough, moderate RD likely increases the viscosity and structural integrity of the aqueous continuous phase (Section 3.1). It hydrates domains, reducing local stress concentration by filling microvoids and improving contact between gluten strands and starch granules, as supported by the observed microstructural continuity and enhanced viscoelastic moduli. These effects can compensate for weaker protein–protein crosslinking, yielding a higher apparent modulus. When RD reached ≥8%, the moduli no longer increased accordingly and tended to lose the earlier improvement, suggesting that excessive RD shifts the system from reinforcement to dilution/segregation of the load-bearing gluten network.

It should be noted that only bulk water-holding capacity was measured in this study. The distribution and mobility of water within different microstructural domains were not directly characterized. This limits a more detailed understanding of RD-induced hydration effects, and future work incorporating techniques such as NMR relaxometry or water activity analysis would be valuable.

#### 3.3.3. Textural and Anti-Staling Properties of Bread with RD

As shown in Table 4 and Figure 5B, breads containing RD (notably 6%) exhibited higher specific volume and reduced crumb hardness and chewiness compared with other samples. At higher RD levels, specific volume gains plateaued and textural advantages diminished. This reflects a transition from a “filling” regime to a “competition” regime. Moderate RD improves continuity and stress transfer, whereas high RD disrupts interfacial cohesion and weakens the gluten framework. During storage, all breads exhibited the typical staling pattern, with increasing hardness and decreasing elasticity (Figure 5C). Compared with the control, RD-containing breads generally showed a slower hardening rate and better retention of viscoelastic properties, indicating delayed staling. The protective effect was most pronounced at moderate RD levels (4–6%).

These macroscopic changes align with the thermal evidence of suppressed starch retrogradation, confirming that RD primarily delays firming by inhibiting starch reassociation in the crumb. Although moisture redistribution contributes to staling, data strongly support a retrogradation-driven mechanism and clarify why the anti-staling effect mirrors the trends in dough rheology and loaf volume: moderate RD optimally modulates hydration and restricts starch chain mobility without inducing disruptive phase separation, thereby preserving gluten continuity and crumb integrity.

## 4. Conclusions

This study shows that RD reshapes wheat starch, gluten, and dough through combined effects of hydration and molecular interactions. In starch, RD competes for water, limits chain mobility, and thus delays gelatinization and suppresses retrogradation. In gluten, RD increases water retention but weakens network integrity, leading to lower disulfide cross-linking and reduced viscoelasticity. Perturbation rheology and structural results suggest that hydrogen bonding is a major contributing interaction in the RD–wheat system.

In the composite dough, moderate RD levels (4–6%) improve matrix continuity and partially offset gluten weakening, which is reflected in better extensibility, gas retention, bread specific volume, and staling resistance. This effect appears to arise from a hydrated, polysaccharide-rich phase, although direct evidence for this structure was not obtained. The findings are based on RD from a single source, and the proposed hydration mechanism remains indirect, so broader validation is still needed.

## Figures and Tables

**Table 1:** Pasting, thermal, and retrogradation properties of wheat starch (WS), as influenced by resistant dextrin (RD) addition.

Properties		0%	2%	4%	6%	8%	10%
PT (°C)		88.1 ± 0.6 ^b^	88.6 ± 0.9 ^ab^	88.9 ± 1.2 ^a^	89.6 ± 0.9 ^a^	89.7 ± 0.6 ^a^	89.7 ± 1.0 ^a^
PV (mPa·s)		1718.0 ± 9.2 ^e^	1822.3 ± 8.4 ^d^	1939.0 ± 8.8 ^c^	1954.6 ± 10.3 ^c^	2074.2 ± 11.8 ^b^	2230.4 ± 13.6 ^a^
TV (mPa·s)		1031.7 ± 8.7 ^e^	1136.4 ± 12.9 ^d^	1192.5 ± 10.6 ^c^	1199.9 ± 9.6 ^c^	1386.2 ± 11.2 ^b^	1586.8 ± 6.0 ^a^
Breakdown (mPa·s)		687.0 ± 6.9 ^c^	689.0 ± 9.2 ^c^	740.0 ± 13.9 ^b^	762.0 ± 12.2 ^a^	688.0 ± 10.7 ^c^	644.0 ± 8.3 ^d^
Setback (mPa·s)		1180.6 ± 13.9 ^a^	1136.4 ± 12.3 ^a^	1168.1 ± 13.4 ^a^	1070.5 ± 12.1 ^b^	1063.0 ± 9.5 ^b^	1014.5 ± 6.3 ^b^
*T_o_* (°C)		57.4 ± 0.3 ^a^	57.3 ± 0.2 ^a^	56.9 ± 0.3 ^a^	57.2 ± 0.2 ^a^	57.0 ± 0.4 ^a^	57.3 ± 0.4 ^a^
*T_p_* (°C)		63.4 ± 0.4 ^d^	63.6 ± 0.3 ^d^	64.3 ± 0.3 ^c^	64.9 ± 0.2 ^b^	66.2 ± 0.2 ^a^	66.4 ± 0.3 ^a^
*T_c_* (°C)		71.2 ± 0.4 ^d^	72.3 ± 0.3 ^c^	72.9 ± 0.5 ^c^	72.8 ± 0.4 ^c^	73.4 ± 0.2 ^b^	74.2 ± 0.3 ^a^
Δ*H*_1_ (J/g)		138.0 ± 0.5 ^d^	140.5 ± 1.0 ^d^	145.0 ± 0.5 ^c^	147.0 ± 0.5 ^b^	149.0 ± 1.0 ^a^	151.0 ± 0.8 ^a^
3 d	Δ*H*_2_ (J/g)	34.0 ± 0.2 ^a^	31.0 ± 0.3 ^b^	30.0 ± 0.5 ^b^	29.0 ± 0.3 ^b^	27.5 ± 0.4 ^bc^	27 ± 0.1 ^c^
	*R* (%)	24.6 ± 0.2 ^a^	22.1 ± 0.3 ^b^	20.0 ± 0.2 ^c^	20.4 ± 0.1 ^c^	18.5 ± 0.3 ^d^	17.9 ± 0.2 ^e^
7 d	Δ*H*_2_ (J/g)	58.0 ± 0.2 ^a^	55.0 ± 0.3 ^a^	51.5 ± 0.1 ^b^	45.0 ± 0.2 ^c^	45.0 ± 0.3 ^c^	43.5 ± 0.2 ^c^
	*R* (%)	42.0 ± 0.1 ^a^	39.1 ± 0.1 ^b^	35.5 ± 0.2 ^c^	31.3 ± 0.1 ^d^	30.2 ± 0.1 ^e^	29.5 ± 0.2 ^f^
14 d	Δ*H*_2_ (J/g)	79.0 ± 0.5 ^a^	76.0 ± 0.5 ^b^	74.0 ± 0.2 ^c^	71.5 ± 0.3 ^d^	73.0 ± 0.3 ^d^	72.5 ± 0.4 ^d^
	*R* (%)	57.2 ± 0.3 ^a^	54.1 ± 0.2 ^b^	51.0 ± 0.2 ^c^	48.0 ± 0.1 ^e^	49.9 ± 0.2 ^d^	48.6 ± 0.3 ^d^

PT, pasting temperature; PV, peak viscosity; TV, through viscosity; *T_o_*, onset temperature; *T_p_*, peak temperature; *T_c_*, conclusion temperature; Δ*H*_1_, gelatinization enthalpy; Δ*H*_2_, retrogradation enthalpy; *R*, retrogradation degree. Different superscript letters in the same column represents significant difference (*p* < 0.05).

**Table 2:** FT-IR band assignments for secondary structure in the amide I region.

Secondary Structure Component	Wavenumber Range (cm^−1^)	Structural Characteristics
α-helix	1650–1660	Intramolecular hydrogen bonding, compact ordered structure
β-sheet	1610–1635, 1680–1695	Intermolecular hydrogen bonding, extended inter-chain structure
β-turn	1660–1680	Reverse chain conformation, connecting secondary structure segments
Random coil	1640–1650	Unordered structure, flexible polypeptide chain conformation

**Table 3:** Gluten and tensile properties of dough with different addition levels of RD.

Parameter	Addition Level of RD (%)					
	0	2	4	6	8	10
Wet gluten (%)	24.2 ± 0.9 ^e^	25.7 ± 0.5 ^d^	27.5 ± 0.7 ^b^	29.6 ± 0.4 ^a^	26.4 ± 0.3 ^c^	19.4 ± 0.5 ^f^
Dry gluten (%)	7.9 ± 0.2 ^a^	7.1 ± 0.1 ^b^	6.5 ± 0.3 ^c^	6.5 ± 0.2 ^c^	6.4 ± 0.3 ^c^	5.7 ± 0.1 ^d^
Water-holding (%)	2.1 ± 0.2 ^d^	2.6 ± 0.1 ^c^	3.2 ± 0.2 ^ab^	3.5 ± 0.2 ^a^	3.1 ± 0.1 ^b^	2.4 ± 0.3 ^c^
Max. tensile resistance (g)	34.3 ± 3.9 ^c^	35.7 ± 1.5 ^c^	35.4 ± 2.7 ^c^	39.4 ± 4.4 ^c^	46.2 ± 2.3 ^b^	59.5 ± 1.5 ^a^
Extensibility (mm)	27.9 ± 2.2 ^a^	27.1 ± 1.1 ^a^	24.5 ± 2.5 ^a^	25.0 ± 1.2 ^a^	22.4 ± 2.3 ^b^	20.7 ± 4.1 ^b^
Tensile work (g·sec)	295.1 ± 3.2 ^c^	316.6 ± 5.1 ^bc^	303.2 ± 7.2 ^c^	341.1 ± 5.2 ^b^	354.6 ± 5.1 ^ab^	378.2 ± 2.3 ^a^

Different superscript letters indicate statistically significant differences among samples (*p* < 0.05).

**Table 4:** Textural properties of bread with RD.

Parameter	Addition Level of RD (%)					
	0	2	4	6	8	10
Hardness (g)	257.92 ± 3.32 ^a^	229.41 ± 7.55 ^b^	167.54 ± 3.84 ^d^	139.89 ± 7.91 ^e^	172.3 ± 3.49 ^d^	187.27 ± 6.35 ^c^
Cohesiveness	0.64 ± 0.003 ^a^	0.62 ± 0.003 ^b^	0.62 ± 0.007 ^bc^	0.61 ± 0.002 ^bc^	0.61 ± 0.002 ^bc^	0.62 ± 0.006 ^bc^
Chewiness (g)	126.67 ± 0.53 ^a^	123.49 ± 2.41 ^b^	105.37 ± 0.79 ^c^	80.84 ± 3.38 ^f^	92.13 ± 1.81 ^e^	95.9 ± 0.88 ^d^
Springiness	0.83 ± 0.005 ^a^	0.81 ± 0.003 ^b^	0.78 ± 0.011 ^d^	0.79 ± 0.009 ^c^	0.79 ± 0.005 ^c^	0.80 ± 0.012 ^bc^
Resilience	0.23 ± 0.005 ^e^	0.26 ± 0.002 ^c^	0.27 ± 0.005 ^b^	0.30 ± 0.06 ^a^	0.26 ± 0.004 ^c^	0.24 ± 0.004 ^d^

Different superscript letters indicate statistically significant differences among samples (*p* < 0.05).

## Data Availability

The original contributions presented in this study are included in the article. Further inquiries can be directed to the corresponding authors.
